# Spinal Metastases in Non-Seminomatous Germ Cell Testicular Tumors: Prognosis and Integrated Therapeutic Approaches—A Systematic Review with an Institutional Case Illustration

**DOI:** 10.3390/curroncol31120551

**Published:** 2024-11-24

**Authors:** Gianluca Scalia, Gianluca Ferini, Zubayer Shams, Francesca Graziano, Giancarlo Ponzo, Eliana Giurato, Maria Grazia Galasso, Vitalinda Pumo, Martina Caruso, Gianluca Galvano, Salvatore Marrone, Jessica Naimo, Giovanni Federico Nicoletti, Giuseppe Emmanuele Umana

**Affiliations:** 1 Neurosurgery Unit, Department of Head and Neck Surgery, Garibaldi Hospital, 95124 Catania, Italy; gianluca.scalia@outlook.it (G.S.); fgraziano@arnasgaribaldi.it (F.G.); gponzo@arnasgaribaldi.it (G.P.); nicolettigiovanni23@gmail.com (G.F.N.); 2 Department of Medicine and Surgery, Kore University of Enna, 94100 Enna, Italy; umana.nch@gmail.com; 3 Department of Radiation Oncology, REM Radioterapia srl, 95029 Viagrande, Italy; 4 Brunel Medical School, Brunel University London, Uxbridge, London UB8 3PH, UK; zubayershams1@gmail.com; 5 Anatomic Pathology Unit, Garibaldi Hospital, 95124 Catania, Italy; egiurato@arnasgaribaldi.it (E.G.); mgalasso@arnasgaribaldi.it (M.G.G.); 6 Oncology Unit, Garibaldi Hospital, 95124 Catania, Italy; vpumo@arnasgaribaldi.it (V.P.); martinacaruso@arnasgaribaldi.it (M.C.); 7 Department of Diagnostic Imaging, Interventional Radiology and Neuroradiology, Garibaldi Hospital, 95124 Catania, Italy; ggalvano@arnasgaribaldi.it; 8 Department of Neurosurgery, Sant’ Elia Hospital, 93100 Caltanissetta, Italy; salvo.mr89@gmail.com; 9 Pain Therapy and Palliative Care Unit, ASP 7 Ragusa, 97100 Ragusa, Italy; jessica.naimo@asp.rg.it; 10 Department of Neurosurgery, Trauma Center, Gamma Knife Center, Cannizzaro Hospital, 95126 Catania, Italy

**Keywords:** non-seminomatous, germ cell tumors, testicular, metastases, spine, chemotherapy, multidisciplinary care, surgery

## Abstract

(1) Background: Testicular cancer, although accounting for only 0.5% to 1% of all solid male cancers, is the most common malignancy in males aged 15 to 35 years. Non-seminomatous germ cell tumors (NSGCT) represent nearly half of all testicular germ cell tumors and are associated with a more aggressive clinical course. Spinal metastases, while rare, pose significant challenges due to their potential to cause spinal cord compression, neurological deficits, and severe pain. This systematic review aims to evaluate prognosis and treatment approaches for spinal metastases in NSGCT, with a focus on multidisciplinary care and treatment outcomes. (2) Methods: A systematic review was conducted following PRISMA guidelines. PubMed, Scopus, and Embase were searched on 18 September 2024, using the Boolean search strategy [(Nonseminomatous germ cell tumor (NSGCT) AND (spinal OR vertebral metastases)]. Case reports, case series, and cohort studies providing detailed patient data were included. Data on patient demographics, tumor histology, metastatic site, treatments, and outcomes were extracted for analysis. (3) Results: A total of 164 cases of NSGCT with spinal metastases were analyzed, with patients aged 23 to 40 years (median: 31.5 years). The lumbar spine was involved in all cases, and spinal cord compression occurred in 59.8% of patients, often causing severe neurological symptoms such as cauda equina syndrome. Chemotherapy, primarily cisplatin-based, was administered in all cases, while surgical interventions, including laminectomy and vertebrectomy, were performed in cases of spinal compression and instability. Complete remission occurred in only 2.4% of patients. Progressive improvement was observed in 56.7% of cases, while 20.1% of patients died. Outcomes varied, highlighting the importance of individualized, multidisciplinary care to manage both systemic and localized disease. (4) Conclusions: Spinal metastases in NSGCT represent a complex clinical scenario, requiring a combination of chemotherapy, surgery, and in some cases, radiotherapy. Chemotherapy remains essential, but surgery is critical for addressing spinal compression and instability. A multidisciplinary approach is vital for optimizing outcomes, as prognosis is variable, with some patients achieving improvement while others face progressive disease or death. Further research is needed to refine the role of radiotherapy and improve long-term treatment strategies for this rare complication.

## 1. Introduction

Testicular cancer, though relatively rare and constituting only 0.5% to 1% of all solid male tumors, is the most common malignancy in males aged 15 to 35 years, with an increasing incidence worldwide, particularly among white males [1,2,3]. Within testicular cancers, non-seminomatous germ cell tumors (NSGCT) account for nearly half of cases and are more aggressive than seminomas, often leading to metastatic disease at the time of diagnosis [4,5,6]. Approximately one-third of patients with NSGCT present with metastases, primarily affecting the lungs, liver, and central nervous system [7,8,9]. Among these metastatic sites, spinal involvement is comparatively rare but represents a uniquely challenging clinical scenario due to the risk of spinal cord compression, neurological compromise, and intractable pain. The complexities associated with spinal metastases in NSGCT stem from the spine’s critical role in mobility and neurological function, making spinal metastases particularly debilitating and difficult to manage.

Clinically, spinal metastases demand a multimodal approach to treatment, balancing systemic control of the disease with localized management of spinal lesions to preserve neurological function and quality of life. Although NSGCT generally has a favorable prognosis, with an overall five-year survival rate of approximately 96%, largely due to high cisplatin sensitivity, outcomes can vary significantly for patients with spinal metastases [10]. The extent of spinal involvement, timely detection, and prompt initiation of treatment are crucial factors impacting survival, neurological outcomes, and symptom control. This systematic review aims to consolidate existing evidence on prognosis and therapeutic strategies specifically for NSGCT patients with spinal metastases. By focusing on survival, neurological preservation, and treatment efficacy, this review highlights the unique challenges and therapeutic complexities encountered in managing spinal metastases in this patient population. Additionally, complementing the systematic review, we also present an illustrative institutional case, offering practical insights into the multidisciplinary management approach employed in a real-world clinical scenario.

## 2. Materials and Methods

### 2.1. Literature Search

A systematic review was conducted according to the Preferred Reporting Items for Systematic Reviews and Meta-Analyses (PRISMA) 2020 guidelines, ensuring a structured and transparent review process [11]. A comprehensive literature search was performed on the PubMed, Scopus, and Embase databases on 18 September 2024. The search strategy employed the following Boolean full-text combination: [(Nonseminomatous germ cell tumor (NSGCT) AND (spinal OR vertebral metastases)]. Given the rarity of the topic, the search aimed to identify not only larger studies but also individual case reports discussing spinal or vertebral metastases secondary to NSGCTs. All relevant studies were exported to Mendeley reference management software, where duplicates were automatically identified and removed. A manual check was then performed to ensure all duplicates were accurately eliminated.

### 2.2. Study Selection

Given the rarity of spinal metastases from testicular NSGCTs, the inclusion and exclusion criteria were broadened to allow for the inclusion of case reports, alongside cohort studies and case series, to ensure comprehensive coverage of the available literature.

Inclusion criteria were as follows:

- Studies involving patients with spinal or vertebral metastases secondary to testicular NSGCTs.
- Articles written in English.
- Case reports, case series, or larger studies with detailed patient data, including demographics, clinical presentation, and treatment outcomes.

**Figure 1:**
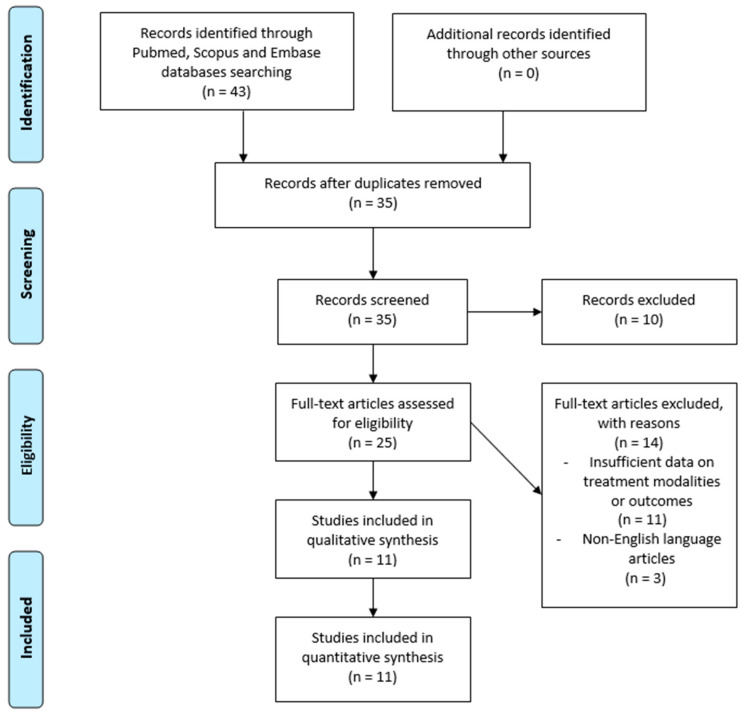
The Preferred Reporting Items for Systematic Reviews and Meta-Analyses (PRISMA) 2020 flow diagram illustrates the study selection process for this systematic review on spinal metastases in non-seminomatous germ cell testicular tumors.

Exclusion criteria applied to:

- Studies involving primary extra-gonadal (e.g., mediastinal, cerebral) NSGCTs.
- Studies focusing on non-spinal/vertebral metastases, such as mediastinal or cerebral metastases.
- Articles lacking original patient data, including narrative reviews, commentaries, or editorials.
- A PRISMA 2020 flow diagram was used to depict the study selection process, beginning with the identification of articles through database searching, the screening of abstracts and titles, and applying the inclusion/exclusion criteria (Figure 1). Additionally, a PRISMA 2020 checklist (Supplementary S1) was completed to ensure comprehensive reporting and adherence to systematic review guidelines. An initial search returned 43 articles. After removing duplicates, 35 unique studies were screened based on title and abstract. Of these, 25 articles underwent full-text review. Ultimately, 11 studies (six case reports and five case series) met the inclusion criteria and were incorporated into the final analysis. These studies were selected based on their relevance and completeness of patient data.

### 2.3. Data Extraction

Data were extracted from each included study using a predefined extraction form, focusing on key patient-related variables (Table 1) [7,8,12,13,14,15,16,17,18,19,20]. The following information was collected:

- Authors and publication year.
- Number of patients described in each study.
- Patient demographics, including age.
- Histological classification of the testicular germ cell tumor (e.g., yolk sac tumor, embryonal carcinoma, teratoma).
- Spinal site of metastasis (cervical, thoracic, lumbar).
- Symptoms associated with spinal metastases (e.g., pain, neurological impairment).
- Surgical interventions, if applicable (e.g., laminectomy, vertebral arthrodesis), along with details of any adjuvant therapies (chemotherapy, radiotherapy).
- Outcomes, including the response to treatment, postoperative recovery, and long-term prognosis.
- Follow-up data, where available, to assess survival duration and recurrence of metastatic disease.

Each dataset was reviewed by two independent reviewers, and any discrepancies were resolved through discussion or arbitration by a third reviewer, ensuring accuracy and consistency in the data extraction process.

## 3. Results

**Figure 2:**
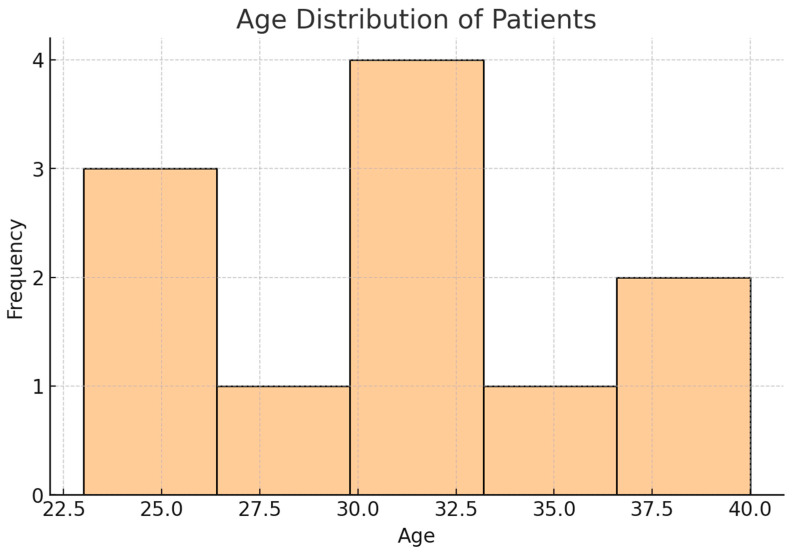
Histogram showing age distribution of patients. This histogram represents the age distribution of patients with NSGCT and spinal metastases, highlighting the concentration of cases within the 23 to 40 range, with a mean age of 31 years.

A total of 164 patients affected by spinal metastases in testicular NSCGTs were included across multiple studies, with ages ranging from 23 to 40 years (Table 2). The mean age of the cohort was 31.0 years, with a median age of 31.5 years and a standard deviation (SD) of 5.66 years, reflecting a moderately young patient population. The age distribution showed an interquartile range (IQR) from 25.75 to 34.0 years, indicating that most patients were in their late twenties to early thirties (Figure 2).

Regarding spinal tumor localization, 100% (164/164) of cases involved the lumbar spine, making it the most affected spinal region. Additionally, 11.6% (19/164) of patients had tumors spanning multiple spinal regions, including the cervical, thoracic, and lumbar spine, indicating more extensive spinal involvement in these cases.

Associated symptoms were primarily characterized by lumbar back pain in 69.5% (114/164) of patients, making it the most prevalent symptom. Spinal cord compression was reported in 59.8% (98/164) of cases, manifesting as a significant clinical issue due to the tumor mass effect. Other notable symptoms included bilateral sciatica, which affected 41.4% (68/164) of patients, and cauda equina syndrome, observed in 8.5% (14/164) of cases, suggesting potential neurological compromise in a subset of the cohort.

Histopathologically, most cases (71.9% (118/164)) did not specify a subtype, indicating a gap in detailed histological reporting across studies. Among the cases with histological details, 16.5% (27/164) were classified as mixed non-seminomatous germ cell tumors (NSGCT) with elements of embryonal carcinoma, choriocarcinoma, and teratoma, while 1.8% (3/164) were identified as malignant teratoma undifferentiated (MTU).

**Figure 3:**
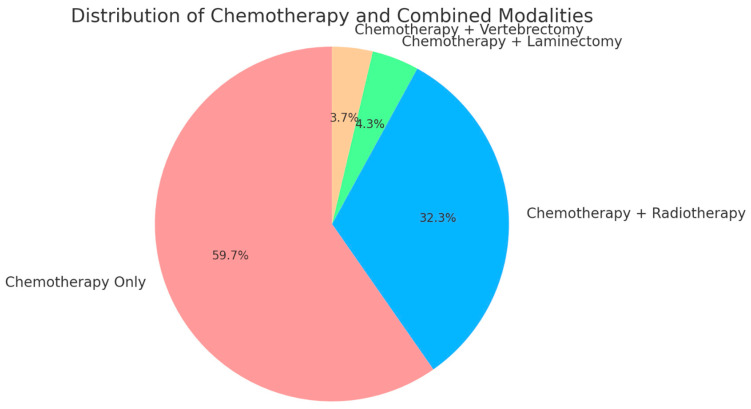
Pie chart showing treatment modalities distribution. This pie chart illustrates the distribution of treatment modalities among patients with NSGCT and spinal metastases. All patients (100%) received chemotherapy, with 32.3% also undergoing radiotherapy, 4.3% receiving chemotherapy in combination with laminectomy, and 3.7% receiving chemotherapy combined with vertebrectomy. The chart clearly depicts chemotherapy as the universal treatment, with various adjunct therapies applied based on individual patient needs.

In terms of treatment modalities, all 164 patients (100%) underwent chemotherapy as part of their therapeutic regimen. Surgical interventions were less frequent but included laminectomy in 4.3% of patients (7/164), and vertebrectomy in 3.7% of cases (6/164). Radiotherapy was utilized in 32.3% (53/164) of cases, typically in combination with chemotherapy, reflecting a multimodal approach to treating these tumors (Figure 3).

The outcomes of these patients varied considerably. Progressive symptomatic improvement was documented in 56.7% (93/164) of cases, suggesting that most patients responded positively to the combination of treatments. Additionally, 59.1% (97/164) of patients exhibited partial responses, demonstrating a degree of tumor control. Stable disease was achieved in 11.6% (19/164) of patients, while complete remission was rare, occurring in only 2.4% (4/164) of cases. Mortality was significant, with 20.1% (33/164) of patients dying from their disease or treatment-related complications. Furthermore, 9.1% (15/164) of patients had no available data on disease outcomes.

The follow-up durations also varied widely, with a mean follow-up period of 19.1 months (SD = 27.6 months), indicating substantial variability in patient monitoring. The median follow-up was notably shorter, at 4 months, with durations ranging from 3 to 93 months. This wide range suggests that while some patients had extended post-treatment surveillance, many were followed for shorter periods, potentially reflecting variability in clinical outcomes or study methodologies.

This cohort analysis of 164 patients with spinal germ cell tumors revealed a predominantly young population, with 100% of cases involving the lumbar spine. Most patients presented with back pain and spinal cord compression, and chemotherapy was the primary treatment across all cases. While many patients showed either improvement or stable disease, complete remission was rare, and mortality remained a significant concern. Follow-up durations varied considerably, reflecting differences in disease progression and the extent of patient monitoring across studies.

This review of NSGCT with spinal metastases highlights the complexity of managing such patients. The frequent involvement of the lumbar spine often results in severe neurological symptoms, requiring a multimodal treatment approach that typically includes chemotherapy and, in some cases, spinal surgery. Although a subset of patients achieves complete remission, others continue to struggle with persistent disease or face mortality. These findings emphasize the importance of multidisciplinary care and long-term follow-up to improve patient outcomes.

### Case Illustration

A 47-year-old male with a two-month history of progressive right testicular swelling and associated discomfort presented to our Department in April 2024. Physical examination and initial evaluation raised the suspicion of a neoplastic process, warranting further diagnostic imaging and evaluation. A total body CT scan and whole-body MRI were subsequently performed, revealing a substantial retroperitoneal mass with evidence of metastatic spread to multiple sites, including the lungs, liver, bones, and regional lymph nodes. The imaging also revealed infiltration of the iliopsoas muscle and thrombosis of the inferior vena cava. These findings, together with elevated serum markers (alpha-fetoprotein and beta-HCG), were highly suggestive of a germ cell tumor.

A CT-guided biopsy of the retroperitoneal lesion confirmed the diagnosis of NSGCT, specifically a yolk sac tumor subtype. The presence of retroperitoneal, hepatic, pulmonary, and osseous metastases, along with vascular involvement, led to the classification of Stage III disease. According to the International Germ Cell Cancer Collaborative Group (IGCCCG) criteria [19], the patient was stratified as having poor prognosis due to the presence of non-pulmonary visceral metastases and markedly elevated tumor markers, including AFP and beta-HCG.

Given the advanced stage and poor prognosis, the patient was started on systemic chemotherapy using the PEB regimen (cisplatin, etoposide, and bleomycin). He completed three cycles of chemotherapy, resulting in a partial response. Follow-up imaging showed a reduction in the size of the primary retroperitoneal lesion, along with partial resolution of some metastatic sites, including those in the lungs and liver. However, despite the favorable oncologic response, the patient developed progressively worsening lumbar pain, which became severe and debilitating.

**Figure 4:**
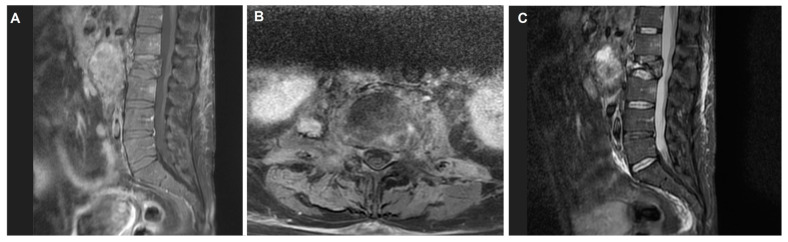
Preoperative lumbar spine MRI showing heterologous lesions at the L1 vertebra with collapse of the L2 vertebral body. (**A**) Sagittal T1-weighted image with contrast enhancement reveals a hypointense lesion in L2 with marked contrast uptake. (**B**) Axial T1-weighted image with contrast highlights irregular enhancement in the L2 vertebra extending into prevertebral structures. (**C**) Sagittal T2-weighted image shows a hyperintense lesion in L2 vertebral collapse with associated signal changes, indicating edema or bone marrow involvement.

This prompted further investigation with a spinal MRI, which identified heterologous lesions at the L2 vertebra with body collapse (Figure 4). These findings suggested metastatic involvement of the spine, raising concerns about potential spinal instability and the risk of neurological compromise. Given these findings, the patient was referred to the neurosurgery department for urgent evaluation and management.

**Figure 5:**
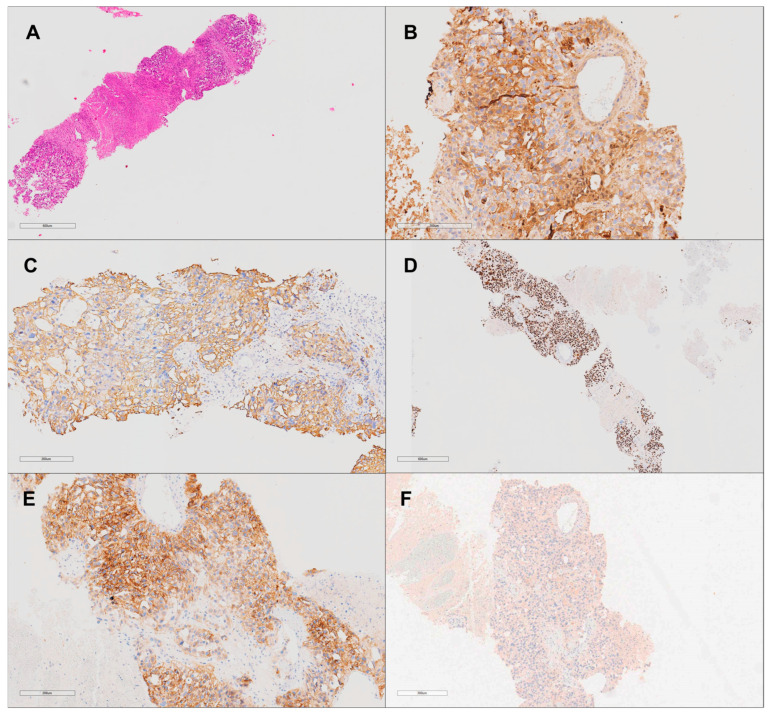
Non-seminomatous germ cell tumor (yolk sac tumor subtype) histological and immunohistochemical analysis at 20× magnification. (**A**) Hematoxylin and eosin stain shows a solid pattern of the yolk sac tumor with adjacent tumoral necrosis. (**B**) Strong positive staining for alpha-fetoprotein (AFP), confirming the diagnosis of yolk sac tumor. (**C**) PANCK immunohistochemistry highlights epithelial components of the tumor. (**D**) SALL4 immunohistochemistry shows nuclear positivity, a marker for germ cell tumors. (**E**) CD117 immunohistochemistry shows focal positivity. (**F**) CD30 immunohistochemistry is negative, excluding embryonal carcinoma.

Upon admission, the patient underwent a vertebral biopsy to further characterize the spinal lesions. Immunohistochemical analysis confirmed that the spinal lesions were metastatic in origin, likely due to contiguous spread from the retroperitoneal mass. The biopsy demonstrated positivity for SALL4, alpha-fetoprotein (AFP), CD117, and panCK, confirming their germ cell origin (Figure 5).

To address the spinal instability and alleviate the patient’s severe pain, the decision was made to proceed with percutaneous vertebral fixation. The surgical procedure involved the placement of titanium transpedicular screws and rods at the Th12, L1, and L3 vertebrae, with the assistance of O-arm imaging and neuronavigation to ensure precision during instrumentation. The primary aim of the surgery was to restore spinal stability, reduce the risk of further vertebral collapse, and improve the patient’s quality of life (QoL). Postoperative imaging confirmed that the hardware was properly placed, with no signs of misalignment, hardware failure, or other complications.

The patient experienced an uneventful postoperative recovery. His lumbar pain improved significantly within the first few days, and his functional mobility began to gradually improve. A multidisciplinary rehabilitation program was initiated, focusing on progressive mobilization and physical therapy aimed at enhancing muscle strength, range of motion, and overall functional independence. Routine blood tests and follow-up imaging demonstrated no postoperative complications, and the surgical wounds healed well without infection or other issues.

At the time of discharge, the patient was in stable condition, with improved pain control and enhanced mobility. He was instructed to continue wearing a thoracolumbar brace during periods of ambulation and standing to provide additional support while his spine continued to heal. In addition, the patient was scheduled for regular follow-up with both the oncology and neurosurgery teams. Continued chemotherapy was planned to address residual disease, and further imaging was arranged to monitor the patient’s response to ongoing treatment. The patient was also referred for outpatient physiotherapy to further assist in his functional recovery.

This case illustrates the complexities of managing a patient with advanced metastatic testicular NSGCT, specifically the yolk sac tumor subtype, which carries a poor-prognosis due to its aggressive behavior and widespread metastasis. The development of spinal metastases in this patient posed a significant clinical challenge, necessitating coordinated care across multiple specialties, including oncology, neurosurgery, and rehabilitation. Despite the aggressive nature of the disease, the patient demonstrated a positive response to chemotherapy and achieved symptomatic relief following spinal stabilization. This case underscores the importance of multidisciplinary care, ongoing long-term monitoring, and tailored interventions in the management of complex oncologic cases involving metastatic spinal disease.

## 4. Discussion

### 4.1. Epidemiology and Clinical Presentation

Spinal metastases in NSGCTs are a rare but serious clinical complication that predominantly affects young males [1,2,3,4]. In the current dataset of 164 patients, the age range spanned from 23 to 40 years, with a mean age of 31.0 years and a median age of 31.5 years, which aligns with the broader epidemiological data identifying testicular cancer as the most common malignancy in men aged 15 to 35 years. This age group is particularly vulnerable to NSGCT, which tends to have a higher metastatic potential than seminomas [21]. Approximately one-third of patients with testicular cancers present with disseminated disease at diagnosis [22], and while spinal metastases are infrequent, their occurrence represents a distinct clinical challenge due to the critical neurological functions supported by the spine [7,8,12,13,14,15,16,17,18,19,20].

In this review, the lumbar spine was involved in 100% of cases, confirming previous observations that the lumbar region is more susceptible to metastases from pelvic and lower abdominal malignancies, including testicular cancers [23]. The frequent involvement of the lumbar spine may be explained by its anatomical proximity to the retroperitoneal lymph nodes, which are a common site of metastatic spread in NSGCT [23]. Notably, 59.8% of patients in this dataset presented with spinal cord compression, a severe condition that can lead to significant neurological deficits, such as cauda equina syndrome (observed in 8.5% of cases) and radiating back pain (reported in 41.4% of cases). These symptoms substantially impact the quality of life (QoL) of affected patients, emphasizing the importance of early intervention to prevent permanent neurological damage.

### 4.2. Therapeutic Approaches and Outcomes

Management of spinal metastases in NSGCTs typically requires a multimodal approach involving systemic chemotherapy, surgical intervention, and, in selected cases, radiotherapy [24,25,26,27]. In this dataset, 100% of patients received chemotherapy, with cisplatin-based regimens (such as BEP: bleomycin, etoposide, cisplatin) forming the cornerstone of treatment [28]. These regimens are highly effective in managing both the primary testicular tumor and its metastatic lesions, including those in the spine. However, chemotherapy alone may not suffice in patients with significant neurological symptoms or spinal instability. In these cases, surgical interventions such as laminectomy (performed in 4.3% of patients) and vertebrectomy (performed in 3.7% of patients) were necessary to decompress the spinal cord and stabilize the spine [18].

Radical inguinal orchiectomy is a surgical procedure essential both for therapeutic reasons (removal of the primary tumor to reduce tumor load and improve systemic treatment efficacy) and diagnostic purposes (providing histopathological insights for accurate diagnosis and treatment planning). In our cohort, except for in five patients, orchiectomy was not explicitly mentioned, which may suggest it was not performed or not reported in the available data. However, the role of orchiectomy remains a standard practice in the management of NSGCT, its usefulness being debated even in cases with metastases [29], and its omission could reflect variability in reporting or the complexity of individual cases.

Surgical decompression, often combined with spinal stabilization, plays a critical role in preventing permanent neurological deficits. The integration of surgery and chemotherapy is vital in addressing both systemic disease and localized spinal complications. For instance, in the case reported by Di Gregorio et al. [18], surgical intervention was necessary to relieve spinal compression and restore structural integrity. Despite the generally favorable prognosis of NSGCT—with five-year survival rates exceeding 90% for early-stage disease—the presence of spinal metastases significantly worsens the outlook. In this dataset, complete remission was achieved in only 2.4% of cases, and 20.1% of patients died due to the disease or treatment-related complications, reflecting the seriousness of spinal metastases.

However, 56.7% of patients experienced progressive improvement, which is encouraging. In addition, 59.1% of the cohort achieved partial responses, underscoring the potential for disease control even in the presence of spinal metastases. The variability in outcomes emphasizes the need for individualized treatment strategies, particularly for patients with extensive metastatic disease.

### 4.3. Role of Radiotherapy

The role of radiotherapy in the management of NSGCT with spinal metastases is less well-established compared to chemotherapy and surgery. Historically, radiotherapy has been used more frequently in seminomas, which are more radiosensitive than NSGCTs; the latter have been less likely to be treated with radiotherapy because of tolerance issues resulting from the higher doses required to control them. However, the huge improvement in radiation delivery with newer techniques may allow further exploration of the use of radiotherapy under safer conditions [30]. Moreover, radiotherapy may still play a role in palliative care, particularly for patients with inoperable metastases or those who are not candidates for surgery due to poor overall health or an extensive disease burden. In this dataset, 32.3% of patients received radiotherapy, often in combination with chemotherapy, to relieve spinal cord compression, reduce tumor mass, and stabilize the spine [31,32].

Radiotherapy may also be particularly useful in anatomically challenging locations, such as the cervical spine, where surgery carries higher risks, and it can be employed postoperatively to manage residual disease following incomplete resection [18]. Nevertheless, the use of radiotherapy must be cautiously balanced against the potential for radiation-induced myelopathy and other rare side effects, especially in younger patients, who may be more prone to long-term complications. Given these risks, further research is needed to define the precise role of radiotherapy in NSGCT with spinal metastases, particularly in combination with other modalities.

### 4.4. Prognostic Factors and Long-Term Outcomes

The prognosis for patients with NSGCT and spinal metastases varies widely, depending on several factors, including tumor burden, response to initial chemotherapy, and the success of surgical interventions [18,33,34,35,36]. 

Serum markers play a crucial role in the management of non-seminomatous germ cell tumors (NSGCT) with spinal metastases, serving as essential tools for diagnosis, monitoring treatment response, and assessing prognosis. Key markers such as alpha-fetoprotein (AFP) and beta-human chorionic gonadotropin (β-HCG) are frequently elevated in NSGCT and correlate with more aggressive disease, offering insights into metastatic potential and overall tumor burden [37]. Elevated levels at diagnosis typically indicate advanced disease and are often associated with poorer outcomes. In patients undergoing chemotherapy, a decrease in these marker levels generally reflects a favorable response to treatment, suggesting tumor regression; conversely, stable or rising levels during treatment may indicate resistance or progression, often prompting more aggressive interventions or treatment adjustments. In cases of spinal metastases, where anatomical complexity may obscure disease progression in imaging, serum markers provide a reliable and minimally invasive means of tracking systemic disease, complementing imaging and physical assessments. Lactate dehydrogenase (LDH), though also used, is less specific and can be influenced by non-cancer-related factors. Elevated serum tumor markers, such as AFP and β-HCG, are frequently associated with poorer prognosis, given their indication of more aggressive disease [37]. In this review, many patients had elevated tumor markers, which correlated with advanced disease. Although only 2.4% of patients achieved complete remission, a substantial proportion (56.7%) demonstrated progressive improvement, suggesting that with effective multimodal treatment, significant disease control is achievable.

The variability in long-term outcomes is particularly notable. In the study by Oing et al. [17], 38% of patients were alive at 18 months, with 28% being disease-free and 9% experiencing measurable disease. These results underscore the potential for long-term survival in some patients, but they also highlight the risk of disease persistence or recurrence. The lack of robust long-term follow-up in many studies complicates the ability to assess overall survival and remission rates accurately, indicating the need for more comprehensive data.

### 4.5. Limitations

Several limitations should be considered when interpreting these results. First, while this review includes 164 patients, the sample size remains small relative to the overall population affected by NSGCT, limiting the generalizability of the findings. Second, there is significant heterogeneity in the clinical outcomes, treatment protocols, and follow-up durations reported in the available literature, making it difficult to determine optimal management strategies with certainty. For example, long-term survival data were frequently incomplete, with many studies failing to provide follow-up beyond 18 months. The retrospective nature of most case reports introduces potential selection bias and reporting bias, as patients with more favorable outcomes may be overrepresented. Additionally, the absence of randomized controlled trials (RCTs) specifically addressing spinal metastases in NSGCT, indeed hindered by their very rare occurrence, further limits the strength of the evidence available to guide treatment decisions.

### 4.6. Future Directions and the Importance of Multidisciplinary Care

The management of spinal metastases in NSGCT requires a multidisciplinary approach, involving oncologists, neurosurgeons, radiologists, and radiation oncologists to ensure comprehensive care. Given the complexity of these cases, early involvement of a multidisciplinary team is critical to optimizing outcomes. Advances in minimally invasive surgical techniques and the development of targeted therapies offer promising avenues for future treatment [38]. Furthermore, the potential role of immunotherapy in NSGCT management, particularly in cases of refractory disease, warrants further investigation [39]. Although still in early research stages, immunotherapy may provide additional options for patients who do not respond to conventional treatments.

Understanding the genetic and molecular underpinnings of NSGCT metastasis could also lead to the development of more effective targeted therapies, potentially improving control of metastatic disease, including spinal involvement [40,41,42,43,44,45,46,47,48,49,50]. Finally, ongoing research into improving spinal stabilization techniques, reducing surgical morbidity, and integrating novel therapeutic agents will be crucial in advancing the management of this challenging patient population.

## 5. Conclusions

Spinal metastases in NSGCT present a complex and challenging condition that requires a comprehensive, multidisciplinary approach for optimal management. While chemotherapy remains central to treatment, surgical interventions are sometimes necessary to address spinal compression and instability, and radiotherapy may be beneficial in selected cases. The coordination between oncologists, neurosurgeons, and radiation specialists is essential to tailor treatments according to the patient’s needs and to manage both systemic and localized disease effectively. A multidisciplinary strategy not only improves symptom control but also enhances the potential for better long-term outcomes. As research progresses, the integration of emerging therapies and collaborative care will be key to advancing treatment strategies and improving survival and quality of life for patients with spinal metastases from NSGCT.

## Figures and Tables

**Table 1:** This table provides a detailed overview of various case reports and studies focusing on patients with non-seminomatous germ cell tumors (NSGCTs) and spinal metastases. It outlines key patient data, including age, spinal site involvement, associated signs and symptoms, histological subtypes, treatment approaches, patient outcomes, and follow-up durations. The table highlights the most affected spinal sites, such as the lumbar region, and summarizes the multimodal treatments used, including chemotherapy, surgery, and radiotherapy. Patient outcomes vary, with some cases showing disease progression or at best stable disease, while others achieved partial response or complete remission.

Authors, Year	Age (Years)	Spinal Site	Associated Signs/Symptoms	Histological Subtypes	Surgery/Adjuvant Therapy	Patient Outcome	Follow-Up (Months)
Hitchins et al., 1988 [12]	39	lumbar (multiple)	Spinal cord compression related symptoms	Malignant teratoma undifferentiated (MTU)	Surgical reconstruction of lumbar spine; Chemotherapy	Disease free	3 months
	25	L2	lumbar back pain	Malignant teratoma undifferentiated (MTU)	Chemotherapy	Progressive improvement	Not specified
Berglund et al., 2006 [13]	25	L2	lumbar back pain and bilateral sciatica	Mixed NSGCT with elements of embryonal carcinoma, choriocarcinoma, and teratoma	Radical right inguinal orchiectomy; laminectomy, anterior corpectomy and spinal fixation in two stages; chemotherapy	Progressive improvement	Not specified
Aldejmah et al., 2007 [14]	29	lumbar	Spinal cord compression related symptoms	Not specified	Left orchiectomy; Chemotherapy	Disease free	Not specified
Grommes et. al., 2011 [15]	(19 patients) median 32	cervical, thoracic and lumbar spine	Spinal cord compression related symptoms and lumbar back pain	Not specified	In 9 patients chemotherapy, 8 patients chemotherapy and radiotherapy, 2 patients palliative care	Progressive improvement	median 26 months
Oechsle, et al., 2012 [16]	(25 patients) median 33	lumbar	lumbar back pain	Mixed NSGCT with elements of embryonal carcinoma, choriocarcinoma, and teratoma	Chemotherapy in all 25 patients; Chemotherapy + radiotherapy in 8 patients	Complete remission in 2%, marker negative remission in 50%, and marker positive remission in 33% of patients. Disease stabilization was achieved in 13% of patients, but one patient (2%) showed progression during high-dose chemotherapy	Median PFS: 11 months Median OS: 24 months
Jamal-Hanjani et al., 2013 [7]	(11 patients) mean 40	lumbar	Spinal cord compression related symptoms; cauda equina syndrome	Not specified	First line chemotherapy; in 4 patients with spinal cord compression, a palliative radiotherapy and a decompressive laminectomy	53% Alive; 32% died	median 18 months
Biebighauser et al., 2017 [8]	37	lumbar, sacrum	cauda equina syndrome	Mixed NSGCT (85% MTU)	Radical right inguinal orchiectomy; Chemotherapy	Stable disease	8 months
Oing et al., 2017 [17]	(65 patients) median 31	lumbar	lumbar back pain and bilateral sciatica	Not specified	Chemotherapy; Radiotherapy in 9 patients	38% Alive; 28% disease free; 9% had stable disease	median 18 months
Di Gregorio et al., 2020 [18]	26	L1	Spinal cord compression related symptoms	Not specified	Right orchiectomy; laminectomy; vertebroplasty; chemotherapy and radiotherapy	Disease progression (CNS involvement)	4 months
Chebli et al., 2022 [19]	23	Th5, L5	cauda equina syndrome	Mixed NSGST	Unilateral left inguinal orchiectomy; Chemotherapy and radiotherapy	Clinical improvement, stable disease	Not specified
Gille et al., 2024 [20]	(37 patients) median 32	lumbar	lumbar back pain and bilateral sciatica	Not specified	Chemotherapy; vertebrectomy (6 patients)	Partial response in 97.5% of cases	median 93 months

**Table 2:** Statistical summary of 164 patients with non-seminomatous germ cell tumors (NSGCT) and spinal metastases. This table includes key patient demographics, treatment modalities, and clinical outcomes. It highlights the involvement of the lumbar spine, frequency of spinal cord compression, and the predominant use of chemotherapy, along with the proportion of patients who underwent surgical interventions and radiotherapy.

Statistical Measure	Value
Total Number of Cases	164
Mean Age of Patients	31.0 years
Median Age of Patients	31.5 years
Age Range	23–40 years
Age Standard Deviation	5.66 years
Number of Lumbar Spine Involvement	164 (100%)
Number of Spinal Cord Compression Cases	98
Percentage of Patients with Spinal Cord Compression	59.8%
Percentage of Patients Treated with Chemotherapy	100%
Percentage of Patients Achieving Complete Remission	2.4%
Percentage of Patients Disease-Free Post-Treatment	Not Specified
Percentage of Alive Patients at 18 Months	38%
Most Common Treatment Modality	Chemotherapy (Cisplatin-based regimens)
Percentage of Patients Treated with Radiotherapy	32.3%
Number of Patients Undergoing Surgical Intervention	Laminectomy (4.3%), Vertebrectomy (3.7%)

## Supplementary Materials

The following supporting information can be downloaded at: https://www.mdpi.com/article/10.3390/curroncol31120551/s1, Supplementary S1: PRISMA 2020 checklist.
